# Effectiveness of a community-based health education intervention on prostate cancer fatalism: a quasi-experimental study

**DOI:** 10.11604/pamj.2024.48.117.34579

**Published:** 2024-07-18

**Authors:** Ruth Gathoni Mbugua, Simon Karanja, Sherry Oluchina

**Affiliations:** 1 Mama Ngina University College, School of Health Sciences, P.O. Box 444-01030, Gatundu, Kenya,; 2 Jomo Kenyatta University of Agriculture and Technology, School of Public Health, Nairobi, Kenya,; 3 Jomo Kenyatta University of Agriculture and Technology, School of Nursing, Nairobi, Kenya

**Keywords:** Prostate cancer, fatalism, screening, Kenya, community-based health education, pessimism, death-inevitability, pre-destination

## Abstract

**Introduction:**

prostate cancer is categorized as the most common cancer in males in 2020 in Kenya at 21.9%. The major challenge with prostate cancer in Low and Middle-Income Countries is the presentation of patients with advanced disease. The rate of prostate cancer screening is low across African countries which has been associated with low knowledge and fatalistic beliefs. The study aimed to assess the effectiveness of community-based health education on prostate cancer fatalism.

**Methods:**

the study design was quasi-experimental. The study was conducted in Kiambu County in the Gatundu North and Kiambu sub-counties in Kenya. A total of 288 men were selected per arm of the study using stratified random sampling. Data were collected using a structured questionnaire at baseline and post-intervention. The intervention was health education through home visits by a Community Health Worker.

**Results:**

in the study, fatalism was associated with prostate cancer screening (P<0.05). There was a significant decrease in prostate cancer fatalism for the attributes of pessimism, pre-determination, and death inevitability in the intervention arm post-intervention. In contrast, in the control arm, there was no significant decrease. Post-intervention, the proportion of respondents with a high perception of fatalism decreased from 51% to 23.6% (P<0.05) in the intervention arm. In contrast, in the control arm, there was no significant decrease.

**Conclusion:**

prostate cancer fatalism significantly influenced prostate cancer screening. Community-based health education significantly reduced pessimism, death inevitability, and pre-destination beliefs about prostate cancer. Tailored culturally relevant health education is an effective strategy to address fatalistic beliefs.

## Introduction

According to the GLOBOCAN 2020 cancer estimates, prostate cancer is the second most frequently diagnosed cancer among men. African men are affected more by prostate cancer in comparison to other men in the world. The mortality from prostate cancer is higher among men in sub-Saharan Africa. Prostate Cancer is categorized as the most common cancer in males in 2020 in Kenya at 21.9% [1]. The reduction in these disparities in mortality due to prostate cancer is dependent on early diagnosis and treatment. The major challenge with prostate cancer in Low and Middle-Income Countries is the presentation of patients with advanced disease that has undergone metastasis. This could be attributed to a lack of knowledge and other barriers to timely diagnosis [1,2]. Prostate cancer screening rates reported across African countries range from 5% to 16% [3-6]. Several barriers to the uptake of screening among African men have been reported which include low knowledge levels and fatalistic beliefs [7]. Fatalistic beliefs are more prevalent among African men in comparison to Caucasian men [8-10]. In Kenya, a significant proportion of men have been reported to hold fatalistic beliefs about prostate cancer [11,12].

Cancer fatalism is an individual's belief that their health results from luck and divine intervention which results in hopelessness and the feeling that they do not influence external events regarding cancer occurrence. The individual has pessimistic beliefs that death is the outcome following a cancer diagnosis [13]. Fatalism is a paramount belief that requires to be considered as it has been associated with a decrease in the propensity of individuals to engage in health promotion and uptake of screening [14]. Individuals with fatalistic beliefs attribute the acquiring of health conditions to fate and less to do with their actions, which may deter their participation in health promotion activities. The disparities in health existing among minority populations have been attributed to fatalistic beliefs [15]. Several studies have investigated the association between fatalism and the adoption of health-promoting behaviors that include the uptake of screening. Fatalistic beliefs have been associated with the under-utilization of prostate cancer screening [11,16-19].

Fatalism has gained much interest, given the disparities that exist in the diagnosis of cancer among individuals of different socio-economic and racial backgrounds. Perception of fatalism has also been associated with poor knowledge of prostate cancer and low education levels among men [18,20,21]. Though fatalism has been investigated among other populations across countries, there exists no similar study to our knowledge among Kenyan men. There is a paucity of studies among African men assessing fatalism and interventions to address this barrier to the uptake of prostate cancer screening. The aim of the study is therefore to assess the effectiveness of community-based health education on prostate cancer fatalism.

**Research questions:** what is the relationship between fatalism and prostate cancer screening? What is the effect of community-based health education on prostate cancer fatalism?

## Methods

**Study design:** this interventional study was quasi-experimental where two sub-counties in Kiambu County were randomly selected as the intervention and control arm of the study. An unpaired design was adopted which entailed the selection of a sample of 288 men aged 40-69 years per arm of the study derived from the Community Health Volunteers (CHVs) registers. A baseline assessment was conducted on both arms of the study using the same tool. This was followed by the participants in the intervention arm receiving health education on prostate cancer delivered by CHVs while those in the control arm did not receive any health education. A post-test was conducted after 6 months to assess the differences in fatalistic beliefs between the intervention and control sites.

**Study area:** the study was conducted in Kiambu County within the central region of Kenya. The intervention arm was Gatundu North sub-county and the control arm was Kiambu sub-county. The two sub-counties were selected to avoid contamination of the study. However, the two sub-counties have similar geographic and socio-economic characteristics. The intervention site had 11 established Community Health Units (CHUs) while the control site had 6 CHUs. The study population comprised 576 consenting men aged 40-69 years residing in the study area in line with the recommended age for screening by the cancer screening guidelines in Kenya [22]. This included 288 men aged 40-69 years per arm of the study. All men already diagnosed with prostate cancer were excluded from the study. A total of 8,504 men aged 40-69 years in the study area were screened for eligibility. Nine participants were excluded from the study due to a decline to participate and failure to meet the inclusion criteria.

**Sampling techniques:** the sample size was determined using the formula for comparing proportions in experimental studies.


$$n=\frac{\left[{\left(Z_{\frac{\alpha }{2}}+Z_{\beta }\right)}^{2}*\left(P_{1}\left(1-P_{1}\right)+P_{2}\left(1-P_{2}\right)\right)\right]}{{\left(P_{1}-P_{2}\right)}^{2}}$$

A total of 288 respondents were selected per arm of the study. Stratified random sampling was applied to select respondents. The primary unit of stratification was a CHU within the study area. The total CHUs were 11 in the intervention arm and 6 in the control arm. The list of households was generated using the Community Health Volunteers (CHVs) register. A table of random numbers was used to select 288 households from the 11 CHUs in the intervention site and 288 households from the 6 CHUs in the control site. Post-intervention: 8 participants were lost to follow in the intervention arm while in the control arm, 1 participant was lost to follow-up as they had relocated from the study area.

**Intervention:** the intervention in the study entailed a 6-month health education program delivered face-to-face by trained CHVs during household visits to the intervention site. A total of 33 CHVs from the established CHUs in the intervention site underwent training on prostate cancer for 2 days using a training guideline based on the Ministry of Health CHVs training Module 13. The training utilized different modes of delivery including interactive lectures, small and large group discussions, demonstration role-play, and return demonstrations. Upon completion of training, every CHV was issued with a CHV tool kit. The tool kit contained key health messages on prostate cancer. This included basic information on the anatomical position of the prostate gland, classification, signs and symptoms, management, and prevention of prostate cancer. The tool kit was applied for reference by the CHVs during the provision of health education during the household visits. The health education delivered to study participants comprised of the initial session and subsequent monthly follow-up visits over 6 months. The principal investigator and the Community Health Assistants supervised the CHVs activities and held monthly meetings in the link health facilities. A post-test was conducted after the 6 months in the intervention and control sites to compare the differences.

**Variables:** the outcome variable in the study was fatalism. Fatalism is an individual's belief that their health is a result of luck, destiny, and divine intervention which results in hopelessness and the feeling that they do not have control over external events related to a cancer occurrence. In the study, fatalism was measured using a modified POWE Fatalism Inventory a validated tool for assessment of fatalism. The tool assessed the four attributes of fatalistic beliefs that included; fear, predestination, pessimism, and death inevitability [13].

**Data collection and analysis:** the data were collected using an interviewer-administered questionnaire from April to October 2019. A team of trained research assistants with a medical background collected data at baseline and 6 months post-intervention in both arms of the study. Before the collection of the data, the questionnaire was pre-tested in Thika sub-county among 29 men. The tool used for data collection was a modified Powe's Fatalism Inventory a validated tool for assessing fatalism [13]. The tool consisted of 11 items that measured four key attributes of fatalism which included; fear, predestination, pessimism, and death inevitability based on a 5-point Likert scale (Strongly Disagree=1 to Strongly Agree=5). The Cronbach's alpha results for prostate cancer fatalism items in the adapted tool used in the current study were reliable (0.87) in measuring the variable. The Statistical Package of Social Sciences Version 22 was used for data analysis. The association of the various attributes of fatalism with the uptake of prostate cancer screening was assessed using Pearson's Chi-square test. Pearson's Chi-square test was used to assess the effect of community-based health education on fatalism. This involved the evaluation of the differences in fatalistic beliefs in the intervention and control arms of the study at baseline and post-intervention. The five-point Likert scale responses were dichotomized to show agreement vs disagreement with the beliefs [23]. The proportions for the various attributes of fatalism (fear, predestination, pessimism, and death inevitability) were then compared in the study arms at baseline and post-intervention using Pearson's Chi-square test. A P value of <0.05 was considered statistically significant at a 95% Confidence Interval.

**Ethical considerations:** ethical approval was sought from the Jomo Kenyatta University of Agriculture and Technology (JKUAT) Institutional Ethics Review Committee (JKU/2/4/896B) and permission was sought from the Ministry of Health, Kenya. The participants' consent was sought after an explanation of the purpose, risks, and benefits of the study through written consent. Participants' confidentiality was ensured and privacy was maintained during the study.

## Results

**Socio-demographic characteristics of the respondents:** at baseline, the response rate was 100% (288) per arm of the study. At post-intervention, 280 respondents in the intervention and 287 respondents in the control arm were interviewed. The socio-demographic characteristics of the participants are shown in Table 1. In the study, the majority of the respondents in the intervention arm were aged 50-59 years while in the control arm, they were aged between 40-49 years. The majority were married at the time of the study. The majority in both arms of the study were affiliated with the Christian religion.

**Table 1:** socio-demographic characteristics

Variable	Category	Control N(%)	Intervention N(%)	Total N (%)	Chi-square/Fishers exact
**Age in years**	40-49	102(35.4)	97 (33.7)	249 (43.2)	P=0.502
	50-59	97 (33.7)	100(34.7)	197 (34.2)	
	60-69	89 (30.9)	95 (31.6)	130 (22.6)	
**Marital status**	Married	227(78.8)	242 (84.0)	469(81.4)	P=0.468
	Single /widowed	61 (21.2)	46(16.0)	107(18.6)	
**Religion**	Christian	283(98.3)	282 (97.9)	565(98.1)	
	Traditionalist	2 (0.7)	4(1.4)	6 (1.0)	Exact= 0.803
	Muslim	3 (1.0)	2 (0.7)	5 (0.9)	
**Education**	None	4(1.4)	2(0.7)	6(1)	
	Primary	89(30.9)	91(31.6)	180(31.3)	P=0.437
	Secondary	151(52.4)	149(51.7)	300(52.1)	
	Tertiary	44(15.3)	46(16.0)	65(15.6)	

Key N= Frequency %= Percentage

**Association of fatalism with prostate cancer screening:** in the study, the fatalism attributes of pessimism, death inevitability, and predestination were significantly associated with prostate cancer screening. The belief that prostate cancer is a predetermined occurrence was associated with screening (P=0.011). The belief of death inevitability following a prostate cancer diagnosis was associated with screening (P=0.031). Pessimistic beliefs about prostate cancer were associated with prostate cancer screening (P=0.036). Notably in the study, the attribute of fear of prostate cancer was not associated with prostate cancer screening (P=0.609) (Table 2).

**Table 2:** association of fatalism and prostate cancer screening

Characteristics	Screened	Never screened	Df	X2	P- value
**I believe if someone was meant to get prostate cancer they will get it as it is God’s will**.					
Disagree	344(93.2)	25(6.8)	1	6.504	0.011
Agree	203(98.1)	4(1.9)			
**I believe if someone got prostate cancer that’s how they were meant to die**.					
Disagree	26(6.1)	403(93.9)	1	3.700	0.026
Agree	3(2.0)	144(98)			
**I believe prostate cancer kills most people who get it**.					
Disagree	22 (6.4)	323 (93.6)	1	3.241	0.031
Agree	3 (3.3)	89 (96.7)			
**If I was diagnosed with prostate cancer, I would not live for more than five years**.					
Disagree	29(3.5)	447(93.9)	1	6.417	0.011
Agree	0(0)	100(100)			
**I think prostate cancer will kill you no matter when it’s found and how it’s treated**.					
Disagree	25(6.3)	370(93.7)	1	4.405	0.036
Agree	4(2.2)	177(97.8)			
**Of all diseases, I am most afraid of cancer**.					
Disagree	5(4.1)	116(95.9)	1	0.261	0.609
Agree	24(5.3)	431(94.7) 431(99.1)			
**I believe if someone gets prostate cancer it’s already too late to get treated for it**.					
Disagree	24(5.4)	418(94.6)	1	0.620	0.141
Agree	5(3.7)	125(96.3)			
**I believe that most people don’t want to know they have prostate cancer due to the fear of dying**					
Disagree	10 (5.7)	166(94.3)	1	0.222	0.638
Agree	19 (4.8)	381(95.2)			
**I believe if somebody gets prostate cancer it doesn’t matter when they find out they will still die**					
Disagree	22(5.9)	350(94.1)	1	1.698	0.198
Agree	7(3.4)	197(96.6)			
**A prostate cancer test will not decrease my chances of dying from prostate cancer**.					
Disagree	21(5.1)	390(94.9)	1	0.017	0.897
Agree	8(4.8)	157(95.2)			
**I believe if someone gets prostate cancer their time to die is near**					
Disagree	25(5.7)	415(94.3)	1	1.632	0.201
Agree	4(97.1)	132(2.9)			

**Effect of community-based health education on prostate cancer fatalism:** there was a significant decrease in the attributes of pessimism, predestination, and death inevitability in the intervention arm post-intervention. The attribute of fear of prostate cancer did not significantly decrease post-intervention. There was a significant decrease in the belief that prostate cancer is a predetermined occurrence in the intervention arm at post-intervention in comparison to the baseline (Table 3). The proportion of respondents who agreed to “I believe if someone was meant to get prostate cancer they will get it as it is God's will” decreased significantly from 37.2% to 18.6% at post-intervention (X^2^=24.318 P≤0.05) while in the control arm, there was no significant change (X^2^=0.659 P=0.417). The proportion of respondents who agreed to “I believe if someone gets cancer that's how they were meant to die” significantly decreased in the intervention arm from 35.4% to 17.9 (X2=22.335 P≤0.05) while in the control arm, there was a significant increase from 31.6% to 39.7% (X^2^=4.136 P=0.042).

There was a decrease in pessimistic beliefs about prostate cancer in the intervention arm post-intervention in comparison to the baseline (Table 3). The proportion who believed that if somebody gets prostate cancer it doesn't matter when they find out they will still die significantly decreased from 39.9% at baseline to 18.2% (X^2^=32.369 P≤0.05) at post-intervention in the intervention arm while in the control arm, there was no significant change (X^2^=0.021 P=0.884). There was a significant decrease from 29.2% to 16.1% in the proportion of respondents who believed that a prostate cancer test would not decrease their chances of dying from prostate cancer in the intervention arm at post-intervention (X^2^=13.870 P≤ 0.05) while in the control arm the proportion increased significantly from 28.1% to 39.2% (X^2^=7.831 P=0.005). Similarly, the proportion of respondents who believed that prostate cancer would kill them no matter when it's found and how it's treated decreased significantly from 37.5% to 21.8% in the intervention arm (X^2^=28.539 P=0.005) while in the control arm, it increased significantly from 25.3% to 38.7% (X^2^=11.736 P≤0.05).

There was a significant decrease in the belief of death inevitability in the intervention arm post-intervention (Table 3). The proportion of respondents who believed that prostate cancer kills most people who get it significantly decreased from 40.3% to 18.2% (X^2^=33.296, P≤ 0.05) in the intervention arm while in the control arm, there was no significant change (X^2^=0.042, P=0.838). Similarly, the proportion of respondents who believed that if they were diagnosed with prostate cancer, they would not live for more than five years decreased from 30.6% to 12.1% (X^2^=28.539, P≤0.05) in the intervention arm while in the control arm, there was a significant increase from 29.9% to 47% (X^2^=17.925 P≤0.05).

**Table 3:** prostate cancer fatalism in the study arms at baseline and post-intervention

	Intervention N (%)			Control N (%)		
Variable	Baseline	Post-intervention	Chi-square	Baseline	Post-intervention	Chi-square
**Perception of Pre-destination towards Prostate Cancer**						
I believe if someone was meant to get prostate cancer they will get it as it is God’s will.	107(37.2)	52(18.6)	24.318 P=<0.05	100(34.7)	109(38)	0.659 P=0.417
If I was diagnosed with prostate cancer, I would not live for more than five years	88(30.6)	34(12.1)	28.539 P=<0.05	86(29.9)	135(47)	17.925 P=<0.05
**Perception of Pessimism towards Prostate Cancer**						
I believe if somebody gets prostate cancer it doesn’t matter when they find out they will still die.	115(39.9)	51(18.2)	32.369 P=<0.05	89(30.9)	90(31.7)	0.021 P= 0.884
A prostate cancer test will not decrease my chances of dying from prostate cancer	84(29.2)	45(16.1)	13.870 P=< 0.05	81(28.1)	112(39.2)	7.831 P=0.005
I think prostate cancer will kill you no matter when it’s found and how it’s treated’	108(37.5)	61(21.8)	28.539 P=0.005	73(25.3)	111(38.7)	11.736 P=<0.05
**Perception of death inevitability towards Prostate Cancer**						
I believe prostate cancer kills most people who get it	16(40.3)	51(18.2)	33.296 P=< 0.05	115(39.9)	117(40.8)	0.042 P=0.838
If I was diagnosed with prostate cancer, I would not live for more than five years	88(30.6)	34(12.1)	28.539 P=<0.05	86(29.9)	135(47)	17.925 P=<0.05

In the study perception of fatalism decreased in the intervention arm post-intervention. The proportion of respondents with a high perception of fatalism decreased from 51% to 23.6%. There was a significant decrease in the perception of fatalism in the intervention arm (X^2^=45.710 P<0.05). In the control arm of the study, the proportion of respondents with high fatalism increased from 58% to 73.2% (X^2^=14.568 P<0.05) (Table 4). There was a significant decrease in prostate cancer fatalism scores for the attributes of pessimism, pre-determination, and death inevitability in the intervention arm post-intervention. In contrast, in the control arm, there was generally no significant decrease in the fatalism scores. The findings of this study suggest that community-based health education delivered by CHVs significantly decreased prostate cancer fatalism.

**Table 4:** comparison of perception of fatalism in the study arms

	Intervention N (%)			Control N (%)		
Variable	Baseline	Post-intervention	Chi-square	Baseline	Post-intervention	Chi-square
**High perception of fatalism**	147(51%)	66(23.6%)	45.71(1)	167(58%)	210(73.2%)	14.568(1)
			P<0.05			P<0.05
**Low perception of fatalism**	141(49%)	214 (76.4%)		121(42%)	77(26.8%)	

## Discussion

Fatalism is a poorly defined phenomenon that makes the designing and implementation of behavioral interventions for cancer prevention and control complex. Fatalistic beliefs are higher in underserved populations and result in decreased participation in cancer-preventive behaviors [13]. In the study, fatalism was associated with prostate cancer screening. Similar findings have been reported in other studies [16,24,25]. Fatalism develops over time in a cyclic pattern whereby men continue observing poor outcomes and deaths from men related to diagnosis with advanced prostate cancer [13]. This contributes further to fatalistic beliefs as with time they develop pessimism towards prostate cancer, perceive helplessness, lose hope, and perceive death as inevitable with a cancer diagnosis. The decrease in fatalism has been predicted to facilitate the participation of men in cancer preventive activities, which include screening for early diagnosis [21].

The study findings indicate that there was a significant decrease in fatalism in the intervention arm in comparison to the control arm at post-intervention. The study findings suggest that community-based health education decreased prostate cancer fatalism attributes of pre-destination, pessimism, and death inevitability. These findings are corroborated in a study where participants in the intervention group who viewed an education video had a greater decrease in colorectal cancer fatalism scores than those in the control group [26]. Similar findings were reported in a study conducted among black men in New York City which assessed the effectiveness of culturally relevant health education on the reduction of fatalism. There was a significant reduction in fatalism following the education intervention [25]. Similarly, a study that utilized a culturally acceptable intervention among African American men, found a significant decrease in fatalism in the intervention group [27]. A similar study in Egypt found a significant decrease in fatalism following an education intervention [28].

Fatalism is a complex barrier to prostate cancer screening that requires critical consideration. Cancer fatalism is prevalent among African men, especially among the underserved populations of low socioeconomic status. The findings of this study support the assertion that men with low levels of knowledge of prostate cancer are more likely to hold fatalistic beliefs. This is evidenced by the significant reduction in fatalism following the education intervention delivered face-to-face by CHVs. Fatalism is a vital factor in the prostate cancer screening decision-making process that requires consideration [25,29]. Designing programs to enhance uptake of cancer screening should address fatalistic beliefs to increase their success [26]. Fatalistic beliefs about prostate cancer may have far-reaching implications that may further worsen the outcomes of prostate cancer treatment and contribute to more deaths as a result of late diagnosis. The provision of culturally relevant health education is urgently needed at the community and facility level to address these beliefs that deter men from engaging in screening.

This study is not without limitations. Randomized Control studies are the gold standard for intervention studies. However, our study was quasi-experimental. Nonetheless, two different sub-counties were selected within the study area, and the study participants were randomly selected. Health education was delivered by several CHWs and to minimize variability in content delivery a standard health education tool kit was used by all the CHWs. The results may be generalized with caution as the study population was predominantly rural.

## Conclusion

Prostate cancer fatalism significantly influenced the uptake of prostate cancer screening. community-based health education significantly reduced pessimism, death inevitability, and pre-destination beliefs about prostate cancer. Tailored culturally relevant health education is an effective strategy to address fatalistic beliefs that deter the uptake of screening in the community.

### What is known about this topic

- *The knowledge of prostate cancer is low among Kenyan men;*
- *The rate of screening for prostate cancer is low among Kenyan men;*
- *A major barrier to the uptake of prostate cancer screening among Kenyan men is the existence of fatalistic beliefs*.

### What this study adds

- *There is the existence of fatalistic beliefs; pessimism, death inevitability, and pre-destination beliefs towards prostate cancer among rural Kenyan men;*
- *The existence of fatalistic beliefs among men influences the uptake of prostate screening;*
- *The increase in knowledge among men through the utilization of community-led education programs may overcome fatalistic beliefs and hence increase the uptake of prostate cancer screening among eligible men*.
